# Incidence of thyroid dysfunction in an Iranian adult population: the predictor role of thyroid autoantibodies: results from a prospective population-based cohort study

**DOI:** 10.1186/s40001-017-0260-2

**Published:** 2017-06-21

**Authors:** Ashraf Aminorroaya, Rokhsareh Meamar, Massoud Amini, Awat Feizi, Azamosadat Tabatabae, Elham Faghih Imani

**Affiliations:** 1 Isfahan Endocrine and Metabolism Research Center, Isfahan University of Medical Sciences, Sedigheh Tahereh Research Complex, Khorram Street, Isfahan, 8187698191 Iran; 2 Isfahan Endocrine and Metabolism Research Center, Isfahan, Iran; 3 Department of Biostatistics and Epidemiology, School of Public Health, Isfahan University of Medical Sciences, Isfahan, Iran

**Keywords:** Hyperthyroidism, Hypothyroidism, Incidence, TPO antibodies, Iran, Thyroid autoantibodies

## Abstract

**Background:**

The prevalence of thyroid dysfunction is high in Isfahan, an area of iodine sufficient in Iran. The aim of this study is to investigate the incidence of thyroid dysfunctions in adults of metropolitan Isfahan and to determine the role of thyroid autoantibodies.

**Methods:**

In a population-based cohort study in 2006–2011, we measured TSH, T4, T3, thyroid peroxidase antibody (TPOAb), and thyroglobulin antibody (TgAb) in 618 out of 2254 people who were euthyroid in 2006. The incidence rates per 1000 person-year (pr) were calculated. The odds ratio (OR), relative risk (RR), and 95% confidence interval (95% CI) were calculated based on logistic regression to quantify the potential predictors of thyroid dysfunction. The receiver-operator characteristic (ROC) analysis along with area under the curve (AUC) was used to determine the optimal cutoff values for baseline TPOAb and TgAb as predictors of thyroid dysfunction.

**Results:**

Within a 6-year follow-up, the incidence rate of hypothyroidism was 3.3 in women and 2.1 in men while the incidence rate of hyperthyroidism was 3.8 in women and none in men per 1000 (person-year). A cutoff value of TPOAb at 38 IU/mL was obtained to differentiate the patients with hypothyroidism and hyperthyroidism, with specificity of 0.75 and sensitivity of 0.76, and AUC (CI 95%) of 0.882 (0.743–1.02), *P* = 0.01 and 0.817 (0.600–1.035) *P* = 0.033, respectively. There is a statistically significant association of hypothyroidism and hyperthyroidism with positive TPOAb [RR (CI 95%): 1.99 (1.27–3.13) and 2.20 (1.23–3.95), respectively].

**Conclusions:**

The incidence rate of thyroid dysfunction is high in Isfahan, and higher TPOAb concentration is its strong predictor.

## Background

Thyroid diseases are the most common endocrine disorders following the diabetes [1]. Thyroid dysfunction is associated with morbidity and deleterious outcomes such as increased risk of coronary artery disease and cardiovascular mortality [2]. These can be decreased dramatically with efficient therapy. Therefore, it is of great interest to understand thyroid disease epidemiology in different regions.

Many cross-sectional studies have been performed to investigate the prevalence of thyroid disorders in various populations [3–5]. However, the numbers of longitudinal cohort studies on thyroid disorders are very limited [4, 6–10]. In cohort surveys, the incidence rates explain a more reliable estimation of newly diagnosed thyroid disorders with valuable consideration for clinicians than the prevalence rates does. In Wickham’s survey over 20-year follow-up, the incidence rate of hypothyroidism was reported 3.5 per 1000 per year (py) and 0.6 per 1000 py in women and men, respectively [8]. The mean incidence of hyperthyroidism was found to be 0.8 per 1000 py in women and negligible in men [8]. The prevalence of newly diagnosed hypothyroidism in two studies of National Health and Nutrition Examination Survey [11] and Colorado study [12] in USA was 4 and 3 per 1000 py, respectively. In a study conducted in Tehran, during 6.7-year follow-up, a significant prevalence rate increase of overall thyroid dysfunction from 1.4% at baseline to 10.5% has been reported [13].

The prevalence and incidence rate of thyroid disorders are associated with several factors including geographic areas, senility, ethnicity, and the amount of iodine intake [8, 14]. Furthermore, the presence of thyroid peroxidase antibody (TPOAb) may act as a biomarker of future thyroid dysfunction [5, 15]. The presence of TPOAb in serum during a median follow-up of 9.1 years has been shown to be associated with increased occurrence of hypothyroidism in the studied population [16]. It is worth noting that thyroid autoantibodies, including TPOAb and thyroid globulin antibody (TgAb), may present in a disease-free population [11]. However, the prevalence of positive thyroid autoantibodies was significantly higher in thyroid dysfunction than in euthyroid subjects [17]. In addition, the level of thyroid autoantibodies varies between populations and may be influenced by heredity, environment, and other factors including birth weight, iodine excess and deficiency, parity, oral contraceptive use, reproductive span, stress, seasonal variation, allergy, smoking, radiation damage to the thyroid gland, viral and bacterial infections [18, 19].

The main aim of the current study is to investigate the incidence of thyroid dysfunction in Isfahani adult population in 2011 who were euthyroid in 2006 in an area, which has been iodine sufficient within last decades [3, 20]. We have investigated the possibility of the thyroid dysfunction prediction with the presence of thyroid autoantibodies and other variables in the population and determined the cutoff point for different levels of TPOAb.

## Methods

### Study population

A population-based cohort study was performed in Isfahan, a metropolitan city located in the center of Iran, from 2006 to 2011. The study is detailed elsewhere [3, 21].

Roughly one-fourth of people (*n* = 618 out of 2254) who were diagnosed with euthyroid in 2006 were followed up. We have lost contacts with the rest of euthyroid population due to the change of addresses. The people gave informed consent and agreed to participate in the study. The ethics committee of the Isfahan University of Medical Sciences approved the protocol for this study according to Declaration of Helsinki.

These patients were invited to the Thyroid Clinic of Isfahan Endocrine and Metabolism Research Center (IEMRC) and were examined by a trained general practitioner and an endocrinologist who were collaborator of the 1st phase of the study in 2006. The same laboratory instruments was used to conduct this study by the same personnel who performed the first phase of the research. All data including demographic, age of menopause, number of parity and abortions, past history of thyroid disorders and smoking, use of medications, especially drugs that interfere with thyroid function, and the past medical history were collected. The patients’ thyroids were also examined physically for goiter or nodule. The height and weight were measured and body mass index (BMI) was calculated by dividing weight (kg) to the height square (m^2^).

### Laboratory measurements

Blood samples were taken to measure T4, T3, TSH, thyroid peroxidase antibody (TPOAb) and thyroid globulin antibody (TgAb), and fasting plasma glucose (FPG) after 8 h of fasting. Serum total T4 and T3 were analyzed by radioimmunoassay (Kavoshyar Co., Tehran, Iran). T4 intra- and inter-assay CV were 4.7 and 4.9%, respectively. Normal range of T4 concentration was 4.5–12.0 μg/dL. T3 intra- and inter-assay CV were 5.2 and 3.9%, respectively. Normal range of T3 concentration was 0.92–2.79 nmol/L.

Serum TSH concentration was assessed by immunoradiometric assay (KavoshyarCo., Tehran, Iran). Intra-assay and inter-assay coefficient of variation (CV) was 1.5 and 1.9%, respectively. The normal range for TSH was 0.3–3.6 mIU/L. Serum TPOAb and TgAb were measured with Rapid ELISA (Genesis Diagnostic Products Corp.). The intra-assay and inter-assay CV for TPOAb were 7 and 5%, respectively. It was less than 12% for TgAb. TPOAb and TgAb concentrations of more than 75 and 100 IU/mL, respectively, were considered as positive. FPG (mg/dL) was measured by photometric method (Pars Azmon kit Lot number: 94011).

Euthyroid was depicted as follows: 0.3 mIU/L ≤ TSH ≤ 3.6 mIU/L, and the definition of hypothyroidism was described as follows: overt (clinical) hypothyroidism (TSH > 10 mIU/L), subclinical hypothyroidism (10 ≥ TSH > 3.6 mIU/L). Definition of hyperthyroidism was described as overt (or clinical) hyperthyroidism (TSH level < 0.1 mIU/L and total T4 > 12 μg/dL and/or total T3 > 2.79 nmol/L) and subclinical hyperthyroidism (TSH < 0.3 mIU/L and total T4 and total T3 within normal range, 4.5–12.0 μg/dL and 0.92–2.79 nmol/L, respectively).

Within 2006–2011, three of people who were euthyroid in 2006 had got thyroid dysfunction and were on treatment. One of them was a known case of overt hypothyroidism. The other two patients were overt hyperthyroidism who had received ^131^I and were known case of postablative hypothyroidism, according to their medical documents. Therefore, results summarized in this report are obtained from 615 participants with no previously known thyroid dysfunction (undiagnosed thyroid function); however, the data gained from these 3 patients are included in the calculation of incidence rate (*n* = 618).

### Statistical analysis

Statistical analyses are performed using statistical package for social science (SPSSversion 15, SPSS, Inc., IL, USA). Normality of quantitative data is evaluated using Kolmogrov–Smirnov and Q–Q plot. Logarithmic transformation is conducted for positive skewed variables. Quantitative and categorical data are expressed as mean (SD) or median (interquartile range) [IQR] and frequency (percentage), respectively. Chi-square test is used for comparing categorical data while analysis of variance (ANOVA) is used for continuous quantitative variables. Crude incidence rate of thyroid dysfunctions per 1000 person-years are calculated in total study sample as well as in different age- and gender-specific groups. To determine the association between TPOAb as independent variable and thyroid dysfunction, we have adopted binary logistic regression analysis in different models. In all analyses, thyroid dysfunction is considered as endpoint. In these analyses, after obtaining relative risk (RR) and 95% confidence interval (95% CI) in crude model, adjustments are made for age, gender, smoking, BMI, positive family history to the first model. Additional adjustment is made for number of parity and abortion, age of menopause, having history of goiter or nodule in the second model. The predictive baseline values of TPOAb levels for different thyroid dysfunctions are evaluated using receiver operating characteristic curve (ROC) analysis, and area under the curve (AUC) and its 95% CI are calculated.

## Results

The total number of participants who were euthyroid in 2006 and enrolled in follow-up study (2006–2011) was 618. Three of the participants were classified as known cases of thyroid dysfunction, one female (46 years old) with overt hypothyroidism on levothyroxine treatment. Two female (57 and 66 years old) patients had known overt hyperthyroidism and were treated with ^131^I.

The mean (SD) age of 615 out of 618 studied population in 2011 is 47.37 (12.02) [range 25–82 years], 314 were men and 301 were women with the average ages of 48.9 (12.08) and 45.88 (11.78) years, respectively (*P* = 0.002). With increasing age, neither the risk of thyroid dysfunction (*P* = 0.1) nor the level of TSH (*P* = 0.26) and the increasing level of autoimmune antibodies (*P* = 0.06) is observed. The risk of thyroid dysfunctions is increased significantly for both women and men, older than 45 years with positive antithyroid antibodies [OR (CI 95%): 2.8 (1.26–6.42), *P* = 0.01, 3.16 (1.17–8.56), *P* = 0.02, respectively]. This risk increased even more when we added menopause as a confounding factor in the model [OR (CI 95%): 3.06 (1.32–7.09 *P* = 0.009)].

The mean (SD) age of menopause (*n* = 86), the number of parity (*n* = 255), and number of abortions (*n* = 78) in 301 women were 48.3 (5.11) years, 3.2 (1.69), and 1.63 (0.95), respectively. The risk of thyroid dysfunctions was higher in menopausal than non-menopausal females [(*n* = 28, 32.6%) vs (*n* = 35, 16.3%), OR (CI 95%): 2.5 (1.39–4.42), *P* = 0.002]. This risk was increased considerably in subclinical hypothyroidism [OR (CI 95%): 2.5 (1.28–4.79), *P* = 0.007].

Clinical nodule and goiter were identified respectively in 5 [in women (*n* = 4, 80%), OR (CI 95%): 4.2 (0.48–38.62), *P* = 0.19] and 92 [in women (*n* = 78, 85%), OR (CI 95%): 7.7 (4.25–13.98), *P* = 0.001] of total 615 participants. The median (range) of T3 and T4 levels were 1.7 μg/dL (0.40–17 nmol/L), 8.1 μg/dL (1.40–56.6 μg/dL), respectively, in whole population. The median (range) of TSH concentration was 2 (0.01–120) mIU/L in whole population, whereas in subclinical hypothyroidism, overt hypothyroidism, subclinical hyperthyroidism, and over hyperthyroidism these were 4.7 (3.7–9.8) mIU/L, 24 (10–120) mIU/L, 0.08 (0.02–0.2) mIU/L, and 0.03 (0.01–0.05) mIU/L, respectively. The overall prevalence of overt hypothyroidism was 2.3% in total and 1.9% in men and 2.8% in women. However, the overall prevalence of overt hyperthyroidism was 1.9% in total, 3.7% in women and no case in men.

Basal characteristics of Isfahani adults with undiagnosed thyroid function (*n* = 615) in 2011 are presented in Table 1. The prominent risk factors for almost all thyroid dysfunctions types were female gender, positive family history of thyroid disease and positive TPOAb (Table 1).

**Table 1:** Basal characteristics of Isfahani adult people (*n* = 615) with newly thyroid function in 2011 were euthyroid in 2006

	Euthyroid, *n* (%)	Subclinical hypothyroidism, *n* (%)	OR (CI 95%)	Overt hypothyroidism, *n* (%)	OR (CI 95%)	Overt + subclinical hypothyroidism, *n* (%)	OR (CI 95%)	Subclinical hyperthyroidism, *n* (%)	OR (CI 95%)	Overt hyperthyroidism, *n* (%)	OR (CI 95%)	Overt + subclinical hyperthyroidism, *n* (%)	OR (CI 95%)
Sex													
Men	281 (54.1)	27 (37.5)	1.96 (1.18–3.26)*	4 (44.4)	1.47 (0.39–5.55)	31 (38.3)	1.90 (1.17–3.07)*	2 (20)	4.7 (0.99–22.45)*	0 (0)		2 (13.3)	7.67 (1.17–34.34)*
Women	238 (45.9)	45 (62.5)		5 (55.6)		50 (61.7)		8 (80)		5 (100)		13 (86.6)	
Age (years)													
20–40	153 (29.7)	14 (19.4)	1	4 (44.4)	1	18 (22.2)	1	4 (40)	1	2 (40)	1	6 (40)	1
40–60	274 (53.2)	42 (58.3)	1.67 (0.88–3.16)	2 (22.2)	0.27 (0.05–1.54)	44 (54.3)	1.36 (0.76–2.44)	5 (50)	0.69 (0.18–2.63)	2 (40)	0.55 (0.07–4.00)	7 (46.7)	0.65 (0.21–1.97)
≥60	88 (17.1)	16 (22.2)	1.98 (0.92–4.26)	3 (33.3)	1.3 (0.28–5.96)	19 (23.5)	1.83 (0.91–3.68)	1 (10)	0.43 (0.04–3.95)	1 (20)	0.86 (0.07–9.7)	2 (13.3)	0.58 (0.11–2.93)
BMI (kg/m^2^)													
<25	151 (29.9)	22 (31.4)	1	2 (22.2)	1	24 (30.4)	1	0 (0)	1	3 (60)	1	3 (20)	1
25–30	230 (45.5)	27 (38.6)	0.8 (0.44–1.46)	3 (33.3)	0.98 (0.16–5.96)	30 (38.0)	0.82 (0.46–1.45)	5 (50)		1 (20)	0.21 (0.02–2.12)	6 (40)	1.31 (0.32–5.33)
≥30	124 (24.6)	21 (30)	1.1 (0.61–2.21)	4 (44.4)	2.43 (0.43–16.51)	25 (31.6)	1.26 (0.69–2.33)	5 (50)		1 (20)	0.40 (0.04–3.95)	6 (40)	2.43 (0.59–9.93)
FPG (mg/d)													
<100	289 (56.2)	51 (70.8)	1	7 (77.8)	1	58 (71.6)	1	7 (77.8)	1	2 (40)	1	9 (64.3)	1
100–126	176 (34.2)	17 (23.6)	0.54 (0.3–0.97)*	1 (11.1)	0.23 (0.02–1.92)	18 (22.2)	0.51 (0.29–0.89)*	2 (22.2)	0.46 (0.09–2.28)	1 (20)	0.82 (0.07–9.12)	3 (21.4)	0.54 (0. 14–2.04)
≥126	49 (9.5)	4 (5.6)	0.46 (0.16–1.33)	1 (11.1)	0.84 (0.10–6.99)	5 (6.2)	0.5 (0.19–1.33)	0 (0)		2 (40)	5.8 (0.81–42.85)	2 (14.3)	1.31 (0.27–6.24)
Smoking													
Yes	42 (8.1)	4 (5.6)	0.66 (0.23–1.92)	1 (11.1)	1.42 (0.17–11.62)	5 (6.1)	0.74 (0.28–1.94)	1 (10)	1.26 (0.15–10.2)	0 (0)		1 (6.7)	0.81 (0.1–6.32)
No	477 (91.9)	68 (94.4)		8 (88.9)		76 (93.9)		9 (90)		5 (100)		14 (93.3)	
Positive family history													
Yes	132 (26.4)	29 (40.8)	1.92 (1.15–3.21)*	2 (22.2)	0.79 (0.16–3.88)	31 (38.8)	1.76 (1.07–2.88)*	7 (77.8)	9.75 (2.0–47.65)*	2 (40)	1.85 (0.30–11.24)	9 (64.3)	5 (1.65–15.24)*
No	368 (73.6)	42 (59.2)		7 (77.8)		49 (61.3)		2 (22.2)		3 (60)		5 (35.7)	
TPOAb (IU/mL)													
Positive	110 (23.4)	27 (42.2)	2.39 (1.39–4.11)*	4 (50)	3.2 (0.8–13.33)	31 (43.1)	2.48 (1.46–4.14)*	8 (80)	13.12 (2.74–62.72)*	4 (80)	13.12 (1.45–118.6)*	12 (80)	13.12 (3.63–47.35)*
TgAb (IU/mL)													
Positive	3 (9.4)	2 (25)	3.22 (0.43–23.65)	1 (100)	0	3 (33.3)	4.83 (0.77–30)	0 (0)		0 (0)		0 (0)	

*BMI* body mass index,* FPG* fasting plasma glucose,* TPOAb* Thyroid peroxidase antibody, as considered positive when level >75 IU/mL,* TgAb* thyroid globulin antibody as considered positive when level >100 IU/mL,* CI * confidence interval

* *P* value <0.05

The incidence of thyroid dysfunctions is reported for whole studied population (*n* = 618) and in different age groups and genders in Table 2. There is a tendency toward higher incidence in older age group (≥60 year old) for all types of thyroid dysfunction, opposed to subclinical hyperthyroidism in which this tendency is toward younger age group (Table 2).

**Table 2:** Incidence of thyroid dysfunction based on sex and age subgroups in Isfahani adult people (*n* = 618) in 2011 who were euthyroid in 2006

Thyroid function status	Sex (*n, *%) incidence		Age (years) (*n*, %) incidence			Total *n* = 618 incidence
	Men	Women	20–40	40–60	≥60	
Overt hypothyroidism	(*n* = 4, 40%) 2.1	(*n* = 6, 60%) 3.3	(*n* = 4, 40%) 3.8	(*n* = 3, 30%) 1.5	(*n* = 3, 30%) 4.6	(*n* = 10, 1.6%) 2.7
Subclinical hypothyroidism	(*n* = 27, 37.5%) 15	(*n* = 45, 62.5%) 26.6	(*n* = 14,19.4%) 13.7	(*n* = 42, 58.3%) 22.8	(*n* = 16, 22.2%) 26.1	(*n* = 72, 11.7%) 20.6
Overt hyperthyroidism	(*n* = 0, 0%)	(*n* = 7, 100%) 3.8	(*n* = 2, 28.6%) 1.9	(*n* = 3, 42.9%) 1.5	(*n* = 2, 28.6%) 3	(*n* = 7, 1.1%) 1.9
Subclinical hyperthyroidism	(*n* = 2, 20%) 1	(*n* = 8, 80%) 4.4	(*n* = 4, 40%) 3.8	(*n* = 5, 50%) 2.5	(*n* = 1, 10%) 1.5	(*n* = 10, 1.6%) 2.7

**Fig. 1:**
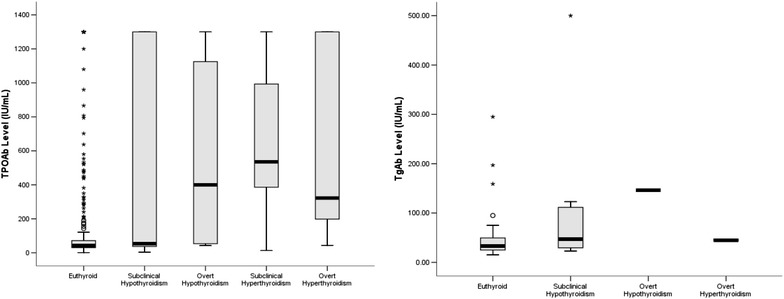
Thyroid peroxidase antibody (TPOAb) and thyroid globulin antibody (TgAb) levels in different thyroid function statuses of Isfahani adult people (*n* = 615) in 2011 who were euthyroid in 2006

TPOAb and TgAb levels were measured in 558 out of 615 and 42 out of 615 participants, respectively. The median (range) of TPOAb and TgAb concentrations were 44.50 (1.30–1300) IU/mL and 34 (15–500) IU/mL, respectively. The TPOAb and TgAb concentrations in different thyroid function statuses are shown in Fig. 1. The median of TPOAb levels in subclinical hypothyroidism, overt hypothyroidism, subclinical hyperthyroidism, and over hyperthyroidism were 53.5 (3.5–1300) IU/mL, 400.5 (42–1300) IU/mL, 465 (13–1300) IU/mL, and 320 (73–1300) IU/mL, respectively. There is a significant difference between TPOAb levels in various thyroid function groups (*P* < 001).

The median of TgAb concentrations in subclinical hypothyroidism, overt hypothyroidism, and overt hyperthyroidism were 62 (23–500) IU/mL, 33 (30–60) IU/mL, and 45 (45–45) IU/mL, respectively. There is no significant difference between various thyroid function groups in terms of TgAb levels (*P* = 0.3). Six out of 42 participants in whom TgAb was measured were positive. Three people (50%) were in euthyroid group and two participants (33.3%) in subclinical hypothyroid group with OR (CI% 95): 3.2 (0.43–23.65), *P* = 0.25 and one patient was with overt hypothyroid (16.7%). Of 558 people, 153 had positive TPOAb [in women (*n* = 91, 59.5%), OR (CI% 95): 1.8 (1.24–2.65), *P* = 0.002].

Table 3 presents the results of multiple binary logistic regression analysis obtained from different models. The regression coefficients suggest positive association of all thyroid dysfunction types to higher levels of TPOAb in different models. In univariate or crude logistic regression analyses, the role of TPOAb level is significant to distinguish between subclinical hypothyroidism (RR = 1.47), overt hypothyroidism (RR = 1.99), subclinical hyperthyroidism (RR = 2.34), and over hyperthyroidism (RR = 2.20) from euthyroid (all, *P* < 0.05). The multivariate logistic regression analyses adjustments were made for some of important potential confounding factors (Models 1 and 2); however, these associations remained statistically significant (*P* < 0.05).

**Table 3:** Relationship between positive thyroid peroxidase antibody (TPOAb) and thyroid dysfunctions in Isfahani adult people with newly thyroid function (*n* = 558 out of 615) in 2011 who were euthyroid in 2006

	Subclinical hypothyroidism RR (CI %95)	Overt hypothyroidism RR (95% CI)	Subclinical hyperthyroidism RR (95% CI)	Overt hyperthyroidism RR (95% CI)
Crude model	1.47 (1.23–1.75)*	1.99 (1.27–3.13)*	2.34 (1.52–3.59)*	2.20 (1.23–3.95)*
Model 1	1.55 (1.28–1.88)*	2.03 (1.27–3.25)*	2.29 (1.37–3.81)*	2.27 (1.17–4.40)*
Model 2	1.56 (1.19–2.05)*	1.25 (0.50–3.14)	4.0 (1.13–14.24)*	

*Crude model* no adjustment was made for confounding variables,* Model 1* adjustment was made for age and sex, smoking, body mass index, positive family history,* Model 2* adjustment was made for age and sex, smoking, Body max index, positive family history, number of parity and abortion, age of menopause, having history of goiter or nodule

* *P* < 0.05.

Receiver operating characteristic (ROC) curve analysis was used to determine the cutoff levels of TPOAb and TgAb in 2006 to predict different thyroid dysfunctions in 2011. The areas under the ROC curves for the thyroid dysfunctions occurrence in relation to thyroid autoimmune antibodies are shown in Table 4. A cutoff value of TPOAb at 38 IU/mL was obtained for differentiating the patients with overt hypothyroidism and hyperthyroidism from euthyroid, with corresponding specificity of 0.75, sensitivity of 0.76, and area under the ROC curve (AUC) (CI 95%) of 0.882 (0.743–1.02), *P* = 0.01 and 0.817 (0.600–1.035) *P* = 0.033, respectively.

**Table 4:** Area under the ROC curve (95% CI) of thyroid dysfunction status according to of thyroid peroxidase antibody (TPOAb) and thyroid globulin antibody (TgAb)

Thyroid function status	TPOAb		TgAb	
	Area (95% CI)	*P* value	Area (95% CI)	*P* value
Overt hypothyroidism	0.882 (0.743–1.02)	0.01	0.809 (0.615–1.0)	0.03
Subclinical hypothyroidism	0.668 (0.537–0.799)	0.01	0.692 (0.572–0.813)	0.004
Subclinical + overt hypothyroidism	0.697 (0.577–0.817)	0.002	0.708 (0.597–0.820)	0.001
Overt hyperthyroidism	0.817 (0.600–1.035)	0.033	0.875 (0.750–1.000)	0.012
Subclinical hyperthyroidism	0.757 (0.573–0.940)	0.056	0.841 (0.720–0.961)	0.011
Subclinical + overt hyperthyroidism	0.784 (0.637–0.931)	0.006	0.856 (0.761–0.951)	0.001

*ROC* receiver operating characteristic, *CI* confidence interval, *TPOAb* thyroid peroxidase antibody, *TgAb* thyroid globulin antibody

## Discussion

This study has estimated the annual incidence of overt hypothyroidism over 6 years to be 2.7 per 1000 and overt hyperthyroidism to be 1.9 per 1000 in Isfahani adults. The incidence of subclinical hypothyroidism and subclinical hyperthyroidism is higher than that of the overt types in both genders after 6 years of follow-up.

We found a positive association between almost all types of thyroid dysfunction and positive TPOAb in different models. The median of TPO and Tg autoantibody levels are increased in various thyroid dysfunctions. The areas under the ROC curves for the occurrence of almost all thyroid dysfunction types are significantly based on TPOAb and TgAb levels in 2006. The optimal cutoff point for TPOAb was 38 IU/mL to predict both overt hypo- and hyperthyroidism.

Recently, an increased incidence rate of thyroid dysfunction in European population has been reported in a meta-analysis to be 2.59 and 2.26 for hypothyroidism and hyperthyroidism, respectively [22]. Majority of studies which estimate incidence rate had been planned in a short period of follow-up [23], while duration of follow-up in Whickham UK cohort study was 20 years (1972 until 1992). In the UK study, the mean annual incidence of spontaneous hypothyroidism was 3.5 and 0.6 per 1000 in women and men, respectively [8]. The incidence rate of overt hypothyroidism in women is similar to our study, 3.5 per 1000 vs 3.3 per 1000. However, we have observed a higher incident rate of 2.1 per 1000 vs 0.6 per 1000 for men. The incidence rate reported for hyperthyroidism in the UK study was 0.8 per 1000 in women with no new cases diagnosed in men while we have identified higher incidence rate of 3.8 per 1000 [8].

Another large population study performed in Scotland, during 9-year follow-up (1993–2001), has shown that the annual incidence of primary hypothyroidism varied between 3.90 and 4.89 per 1000 in women and between 0.65 and 1.01 per 1000 in men [4, 24]. For hyperthyroidism, the annual incidence rate was 0.77/1000 in women and 0.14/1000 in men [24]. In a large population study in Sweden, the reported incidence for overt hyperthyroidism varied between 0.4 per 1000 in women and 0.1 per 1000 in men [7]. Although, the incidence rates of hypothyroidism reported in large population studies are similar to our findings, the hyperthyroidism incidence determined in our study is higher than previous reports. This difference of incidence rate in hyperthyroidism is originated from the difference between the overall median ages of these studies. The peak age of incidence rate for Graves’ disease is between 20 and 49 years [25, 26] and the overall median age of current study is 47 years, while this median is 58 in Whickham Survey [8] and 61.8-year old in study conducted in Denmark [27].

Following the salt iodization in recent decades in the whole country, Iran has been considered as an iodine-sufficient area [3, 20, 28]; therefore, the increased iodine intake may have been contributing to autoimmune thyroid disease induction [29]. Following the addition of iodine to salt nutrition in Austrian multi-center retrospective study, the incidence rates of overt and subclinical hyperthyroidism are increased by 36 and 64%, respectively [30]. The incidence rate of hyperthyroidism has increased every year following salt iodization in Denmark, a moderately iodine-deficient area [31]. Iodide stimulates thyroid follicular cells and induces chemokine upregulation leading to thyroid autoimmune disease [32]. The higher incidence rate of hypothyroidism in men in our study compared to previous studies, 2.1 per 1000 vs 0.6–1.01 per 1000, and a strong correlation between TPOAb and thyroid dysfunctions might be associated with the environmental factors that promote autoimmunity in our population, such as iodine supplement.

The incidence of thyrotoxicosis is shown to rise in women but not in men in a large population study conducted in Tayside, UK [4], similar to our study. However, the higher incidence rate has been observed in women more than men for all types of thyroid dysfunction.

A cohort study conducted in 1999 in Tehran during 6.7-year follow-up showed the incidence rate of hypothyroidism in 1000 subjects py in women and men to be 0.28 and 0.21, respectively, while hyperthyroidism incident rate was 1.4 and 0.21 in 1000 subjects py in women and men, respectively [13]. The incidence of both overt hypothyroidism and hyperthyroidisms in the present report is much higher than the study conducted in Tehran, but hyperthyroidism in men is similar (none vs 0.2 per 1000) [13]. This difference could be originated from different sample sizes, definition for thyroid dysfunction, time of study (1999–2006 vs 2006–2011), and possible unknown environmental factors [13]. The duration of follow-up in both studies is approximately equal (6 vs 6.7 years) and similar differences are observed in the prevalence of thyroid dysfunction in both studies overt hypothyroidism (2.8 vs 0.28 in women and 1.9 vs 0.21 in men) and overt hyperthyroidism (5.2 vs 1.4 in women and no case vs 0.21 in men), respectively [13].

Some variables including positive antithyroid antibodies, positive family history, age, race, and gender have been shown to be correlated to the thyroid dysfunction [7, 8, 11]. The incidence rate of thyroid dysfunction is increased roughly two to eight times with age and female gender in the UK and Australia [7, 33], similar to our findings. The Wickham study shows that the positive antithyroid antibodies and serum TSH levels have markedly increased only in women older than 45 years but not in men [8]. This is in agreement with our finding that increased risk of thyroid dysfunctions is appeared to be higher in women, specifically those who are older than 45 years. The positive family history of thyroid diseases is unassociated with increased odds of developing overt hypothyroidism [8]. However, our results indicate that overt hyperthyroidism is strongly associated with family history of thyroid diseases.

Positive thyroid autoantibodies might be a risk factor for developing abnormal thyroid function [8]. Both rising level of TSH and the presence of autoimmune antibody have been identified with age [11], opposed to our study. This might be originated from the race differences between two studies and lower number of the older age group participated in the current research.

Majority of studies have adopted the presence or absence of antithyroid antibodies, the age, and gender of the studied population to predict progression from euthyroidism to overt or subclinical hypo- and hyperthyroidism [16, 34]. However, few long-term follow-up studies, 13-year follow-up in Australia [35] and 20-year follow-up in the UK [8], have associated the increased level of thyroid antibodies (TPOAb or TgAb) to raised risk of hypothyroidism and increased levels of serum TSH [8, 35]. The positive rate for TPOAb and/or TgAb is shown to be 13.8% in euthyroid population in China [17], while this rate is much higher in our study (27%). This could be attributed to higher incidence rate of hyperthyroidism in our population than other countries.

Consistent with previous researches [11, 17, 36], the positive thyroid autoantibodies show substantial correlation with female gender and developing of almost all thyroid dysfunction types in the present report. TPOAb was identified as a major autoantigen. It is also the main enzyme in thyroid hormone synthesis [37]. Many studies have shown this relationship between TPOAb and thyroid dysfunction but the cause and effect is still unclear [38, 39]. The presence of TPOAb might be correlated with severity of thyroid lymphocytic infiltration, despite the presence or absence of hypothyroidism [40, 41].

Both induced hypothyroidism and hyperthyroidism by autoimmune thyroid diseases have been associated with positive TPO level [42]. Increased serum TPOAb level is the first sign of progression to hypothyroidism in healthy individuals as a consequence of Hashimoto’s thyroiditis [43]. In the present study, we adjusted many variables to determine the correlation between TPOAb and thyroid dysfunctions. The relationship remained positive after adjustments in almost all types of thyroid dysfunctions. This is in agreement with previous study that identified both TPOAbs and TSH level as independent predictors for future hypothyroidism [16].

A positive correlation has been shown between the number of parity and development of autoimmune thyroiditis [44]. On the other hand, the increased level of TPOAb has been a significant risk factor for abortion, prematurity [45, 46], and higher age at menopause [47]. The marked changes in the amounts of estrogen secretion might also affect the subsequent development of thyroid autoimmunity [48]. In previous prolonged follow-up studies, in agreement with our findings, TSH and thyroid autoantibody levels in pre- and postmenopausal women have insignificant differences [48, 49]. On the other hand, late menopause and earlier menarche are associated with a higher risk of Hashimoto’s thyroiditis [50], suggesting that the reproductive span might be of importance.

In addition, the family history of goiter predisposes patients to the presence of autoantibodies in the serum [51, 52]. Our findings show a prominent relationship not only between thyroid dysfunctions and positive TPOAb but also with TPOAb concentration. After adjustment with the number of parity, abortion, age of menopause, history of goiter or thyroid nodules, this correlation became even stronger.

A longitudinal study in Australia, with 13-year follow-up, calculated cutoff point of 29 IU/mL for TPOAb in predicting hypothyroidism [35]. A similar study in Tehran on healthy euthyroid individuals with 10-year follow-up presented the optimal cutoff point for TPOAb of 18.38 IU/mL for predicting overt hypothyroidism [53]. The cutoff point of TPOAb for predicting overt hypo- and hyperthyroidism in our study is higher (38 IU/mL) than these reports. However, our value is very close to the Busselton thyroid study in Australia, where the upper limit of the reference interval for TPOAb is reported to be less than 35 IU/mL [54]. The National Academy of Clinical Biochemistry also reports the reference interval of 30 IU/mL for upper limits of TPOAb [43]. These differences in TPOAb reference limits are most probably originated from different experimental methods, iodine intake, genetic factors, and characteristics of the populations such as gender and age [11, 55, 56]. We also believe that different levels of sensitivity adopted by researchers in various studies may result in discrepancy between cutoff point of TPOAb for predicting thyroid dysfunctions.

The incidence rate of thyroid dysfunction in our study is higher than the reported incidence rate of the world. TPOAb positivity is strong predictor for this incidence rate. The optimal cutoff point for TPOAb in a sample of Isfahani population is slightly higher than previous studies.

## Conclusions

The prevalence of thyroid dysfunction is studied in Isfahan, an area of iodine sufficient in Iran. We have found that the incidence rate of thyroid dysfunction in our study is higher than previous similar reports, specifically within the hyperthyroidism cases. Positive TPOAb and higher TPOAb concentration are suggested as strong predictors for thyroid dysfunction in Isfahan.

## Abbreviations

py: per year
TPOAb: thyroid peroxidase antibody
TgAb: thyroglobulin antibody
IEMRC: Isfahan Endocrine and Metabolism Research Center
BMI: body mass index
FPG: fasting plasma glucose
CV: coefficient of variation
RR: relative risk
95% CI: 95% confidence interval
ROC: receiver operating characteristic curve
AUC: area under the curve
